# Identification of novel and conserved miRNAs involved in pollen development in *Brassica campestris* ssp. *chinensi*s by high-throughput sequencing and degradome analysis

**DOI:** 10.1186/1471-2164-15-146

**Published:** 2014-02-21

**Authors:** Jianxia Jiang, Meiling Lv, Ying Liang, Zhiming Ma, Jiashu Cao

**Affiliations:** 1 Laboratory of Cell and Molecular Biology, Institute of Vegetable Science, Zhejiang University, Hangzhou, 310058, China

**Keywords:** *Brassica campestris*, *Brassica rapa*, miRNAs, Pollen development, High-throughput sequencing, Deep sequencing, Degradome analysis

## Abstract

**Background:**

microRNAs (miRNAs) are endogenous, noncoding, small RNAs that have essential regulatory functions in plant growth, development, and stress response processes. However, limited information is available about their functions in sexual reproduction of flowering plants. Pollen development is an important process in the life cycle of a flowering plant and is a major factor that affects the yield and quality of crop seeds.

**Results:**

This study aims to identify miRNAs involved in pollen development. Two independent small RNA libraries were constructed from the flower buds of the male sterile line (*Bcajh97-01A*) and male fertile line (*Bcajh97-01B*) of *Brassica campestris* ssp. *chinensis*. The libraries were subjected to high-throughput sequencing by using the Illumina Solexa system. Eight novel miRNAs on the other arm of known pre-miRNAs, 54 new conserved miRNAs, and 8 novel miRNA members were identified. Twenty-five pairs of novel miRNA/miRNA* were found. Among all the identified miRNAs, 18 differentially expressed miRNAs with over two-fold change between flower buds of male sterile line (*Bcajh97-01A*) and male fertile line (*Bcajh97-01B*) were identified. qRT-PCR analysis revealed that most of the differentially expressed miRNAs were preferentially expressed in flower buds of the male fertile line (*Bcajh97-01B*). Degradome analysis showed that a total of 15 genes were predicted to be the targets of seven miRNAs.

**Conclusions:**

Our findings provide an overview of potential miRNAs involved in pollen development and interactions between miRNAs and their corresponding targets, which may provide important clues on the function of miRNAs in pollen development.

## Background

Pollen, as the male gametophyte, participates in sexual reproduction in angiosperms and influences seed yield and quality. Pollen development is one of the most fascinating and critical processes that are critical for successful reproduction in the life cycle of flowering plants [1-3]. By employing a variety of resources and novel techniques, scientists have made significant progress in pollen research toward a deeper understanding of pollen development. In 2010, the phytohormone brassinosteroid was verified to control male fertility by regulating the expression of key genes involved in *Arabidopsis* anther and pollen development, such as *SPL*/*NZZ*, *EMS1*/*EXS*, *DYT1*, *TDF1*, *AtMYB103*, *AMS*, *MS1*, and *MS2*[4]. Honys and Twell conducted a genome-wide study on pollen transcriptome in *Arabidopsis* based on microarray analysis. Over 3500 genes were predicted to be expressed in pollen, out of which more than 1400 genes were pollen-specific [5]. A large-scale genetic screen was conducted in *Arabidopsis*, and a number of genes were identified to be involved in pollen exine production, including fatty acid ω-hydroxylase *CYP704B1*, putative glycosyl transferases At1g27600 and At1g33430, 4-coumarate-coenzyme A ligase *4CL3*, and polygalacturonase *QUARTET3*[6]*.* Until now, a series of genes which were successively renamed *Brassica campestris Male Fertile (BcMF) 2*[7], *BcMF3*[8]*, BcMF4*[9], up to *BcMF21*[10]*,* have been demonstrated to be involved in pollen development in *Brassica campestris*. For example, among these genes, one type was polygalacturonase gene, which participated in pollen extine or intine development, such as *BcMF6*[11], *BcMF2*[7], and *BcMF9*[12]. These previous studies provide important information for understanding the gene regulatory networks of pollen development. However, the mechanisms that underlie pollen development remain unclear, and further studies must be conducted.

miRNAs are a newly identified class of endogenous non-coding small RNAs that have become a research hotspot because of their negative regulatory function in gene expression at the posttranscriptional level by degrading target mRNAs or repressing gene translation. Numerous studies have previously shown that miRNAs have important functions in regulating a wide range of plant developmental processes, including lateral root development [13], leaf development and polarity [14], vegetative phase change [15], flowering time and floral organ identity [16,17], plant nutrient homeostasis [18], signal transmission [19], and response to environmental biotic and abiotic stresses [20,21]. Among miRNAs that are known to function in a variety of plant development processes, few miRNAs in pollen tissue have been reported. Wei et al. identified 292 known miRNAs and 75 novel miRNAs in *Oryza sativa*. A total of 103 out of the 292 known miRNAs were enriched in developing pollen, and more than half of the 75 novel miRNAs displayed pollen- or stage-specific expression [22]. With the use of microarray, Chambers and Shuai detected 26 miRNAs which showed significant differences in expression between mature pollen and inflorescence in *Arabidopsis.* They confirmed the expression of 22 miRNAs in mature pollen by using real-time PCR, with most of miRNAs being expressed in low abundance [23]. Grant-Downton et al. detected 33 different microRNA families in mature pollen, and several showed pollen-enriched expression compared with leaves, such as miR156, miR2939, miR158, and miR845 [24]. Previous studies demonstrated the existence of miRNAs in pollen or inflorescence. However, studies must be conducted to investigate whether miRNAs participate in the pollen development and to determine their corresponding functions.

In this study, two small RNA libraries were constructed from the flower buds of the sterile line *Bcajh97-01A* (A line) and the fertile line *Bcajh97-01B* (B line) plants. A total of 24 known miRNAs were detected, 54 conserved miRNAs and 25 pairs of novel miRNA/miRNA* were identified. Meanwhile, 18 differentially expressed miRNAs were identified by comparing their expression abundances in the two libraries. Results from qRT-PCR agreed with those from high-throughput sequencing. To search for the target genes of identified miRNAs, degradome sequencing was conducted with the inflorescences of the B line plants. A total of 15 targets were predicted to be cleaved by seven miRNAs. Our study provides clues to explore miRNA group involved in pollen development and the interactions between miRNAs and their targets.

## Results

### Analysis of small RNA library data sets and the small RNA profile

**Figure 1:**
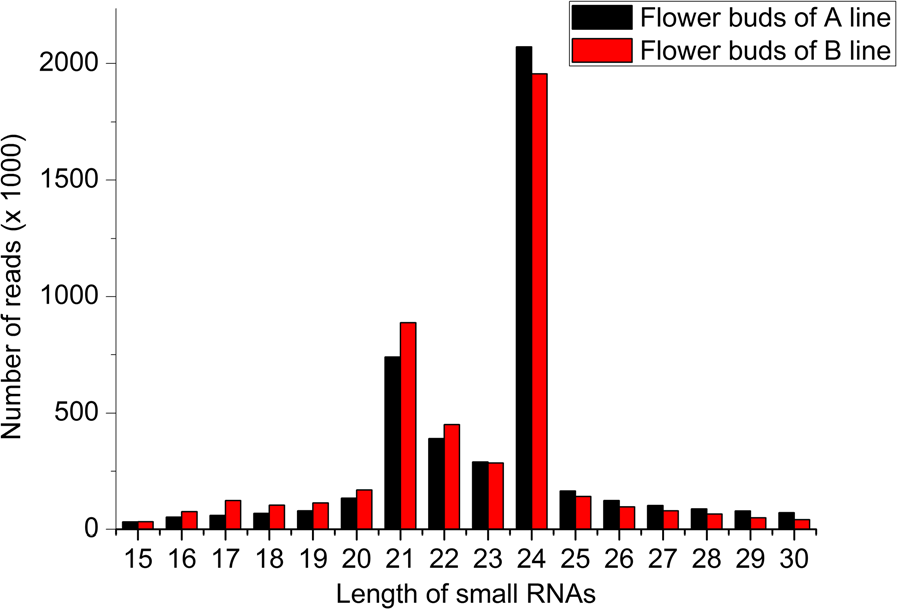
**Length distribution of small RNAs in flower buds of A line and B line libraries of *Brassica campestris.***

To identify miRNAs involved in pollen development in the male sterile line ‘*Bcajh97-01A*’ and the male fertile line ‘*Bcajh97-01B*’, two independent small RNA libraries from the flower buds collected from the two lines were sequenced by using Illumina Solexa high-throughput sequencing technology. The two libraries generated a total of 6998586 and 6792888 raw reads, respectively (Table 1). The raw reads of the two libraries were uploaded to SRA database of NCBI and two accession numbers were obtained, which were SRX462325 and SRX464860. After removing the reads because 3ADT was not found, reads <15 bases, and junk reads, 5098547 and 5309119 sequences were obtained with lengths that range from 15 nt to 30 nt, respectively. After further filtering the RFam (rRNA, tRNA, snRNA, snoRNA, and other Rfam RNAs) and Repbase sequences, a total of 4540620 and 4667617 mappable small RNA sequences, respectively, were obtained (Table 1). The length distributions of small RNAs were very similar between the two libraries (Figure 1). In general, the majority of the small RNAs ranged from 21 nt to 24 nt in size. The 24 nt small RNAs in total sequence reads were the most dominant, followed by 21 nt small RNAs (Figure 1).

**Table 1:** **Analysis of small RNA sequences from the flower buds of A line and B line of *Brassica campestris***

**Category**	**Flower buds of A line**		**Flower buds of B line**	
	**Sequences**	**Unique sequences**	**Sequences**	**Unique sequences**
Raw reads	6998586	2093851	6792888	2463600
Number of reads removed because 3ADT was not found	507331	/	212884	/
Number of reads removed because of <15 bases	1365432	/	1237678	/
Junk reads	27276	/	33207	/
RFam	541924	56567	611768	76694
Repbase	35231	8193	73970	13031
Mappable sequences	4540620	1696791	4667617	2075669

### Identification of known miRNAs

To identify known miRNAs in *Brassica campestris*, all mappable small RNA sequences were compared with the known plant miRNAs in the miRBase database. A total of 24 small RNAs that have the same sequences with the known bra-miRNAs in miRBase were identified (i.e., 24 known *Brassica campestris* miRNAs were identified). The numbers of reads of the 24 known miRNAs in the two small RNA libraries from flower buds of A line and B line are listed in Additional file 1: Table S2 Among the 24 known miRNAs, bra-miR159a, bra-miR160a-5p, bra-miR171e, bra-miR1885b, and bra-miR5724 showed very high expression levels.

### Novel miRNA on the other arm of known pre-miRNA

The advent of high-throughput sequencing technology has given rise to the discovery that a great number of miRNAs and miRNAs* are simultaneously present on two arms of pre-miRNA secondary structures. miRNA and miRNA* are renamed miRNA-3p or miRNA-5p, which indicates their locations in the 5′ arm or 3′ arm of pre-miRNA secondary structures. Through high-throughput sequencing, eight novel miRNAs on the other arm of known pre-miRNAs were identified. miRNA sequences and the corresponding number of reads in flower buds of the A line and B line are listed in Table 2.

**Table 2:** Identification of novel miRNAs on the other arm of known pre-miRNAs

**miR_name**	**miR_seq**	**Len**	**Flower buds ** **of A line**	**Flower buds ** **of B line**
bra-miR164a-p3	CACGTGCTCCACTCCTCCAAC	21	108	44
bra-miR167b-p3	GATCATGTTCGCAGTTTCACC	21	6,061	3,762
bra-miR171b-p5	AGATATTAGTGCGGTTCAATC	21	53	17
bra-miR171e-p5	TATTGGCCTGGTTCACTCAGA	21	200	1,049
bra-miR172a-p5	GCAGCACCATCAAGATTCACA	21	8	8
bra-miR824-p3	CCTTCTCATCGATGGTCTAGA	21	520	3,882
bra-miR1140-p5	TCCGATTGGCTTTAGGCTGTTG	22	709	1,068
bra-miR5718-p5	TGTTCTGGTTTGATTTTGAAC	21	1,686	1,258

### Identification of new conserved miRNA families and new miRNA members

To identify conserved miRNAs in *Brassica campestris*, all mappable small RNAs were mapped to the *Brassica campestris* genome sequences and known plant miRNAs in miRBase. If the small RNAs can exactly map to *Brassica campestris* genome sequences and can also match known plant miRNAs with no more than three mismatches, these small RNAs were classified as candidate conserved miRNAs. Five criteria described in the materials and methods were mainly used to strictly screen the candidate conserved miRNAs. As a result, 54 miRNAs (27 pairs of miRNAs) that belong to 15 families were identified (Additional file 1: Table S3). These 54 miRNAs have not been previously reported as bra-miRNAs in miRBase and show high sequence similarity to some of the known plant miRNAs. Most of these miRNAs were 21 nt long, with only few miRNAs having lengths of 20, 22, or 23 nt; this characteristic is common in most plant species. For the bra-miR156 family, eight members were obtained by using deep sequencing. Two family members (bra-miR168a and bra-miR168b) of bra-miR168 were identified. Two pairs of miRNAs that belong to bra-miR168a, with one pair being bra-miR168a-1-p5 and bra-miR168a-1-p3, the other pair being bra-miR168a-2-p5 and bra-miR162a-2-p3, were identified. The two pairs of bra-miR168a shared the same mature sequences. However, they were from different precursors, i.e., they came from different loci of the *Brassica campestris* genome. The two precursor sequences of bra-miR168a were highly similar with each other. These two pairs of miRNAs were called sub-members. This type of sub-member was also observed in the bra-miR395 family. Four sub-members (bra-miR395a-1, bra-miR395a-2, bra-miR395a-3, bra-miR395a-4) were identified for bra-miR395a. This phenomenon suggests that some highly similar *MIRNA* gene might be produced by a replication event from one origin sequence to another one, which results in more copies of the miRNA group. Except the abovementioned three miRNA families, only one miRNA member was identified for the rest of miRNA family. At the same time, six new miRNA members that belonged to three known miRNA families were discovered in the two small RNA libraries (Additional file 1: Table S4).

### Identification of novel miRNAs

**Figure 2:**
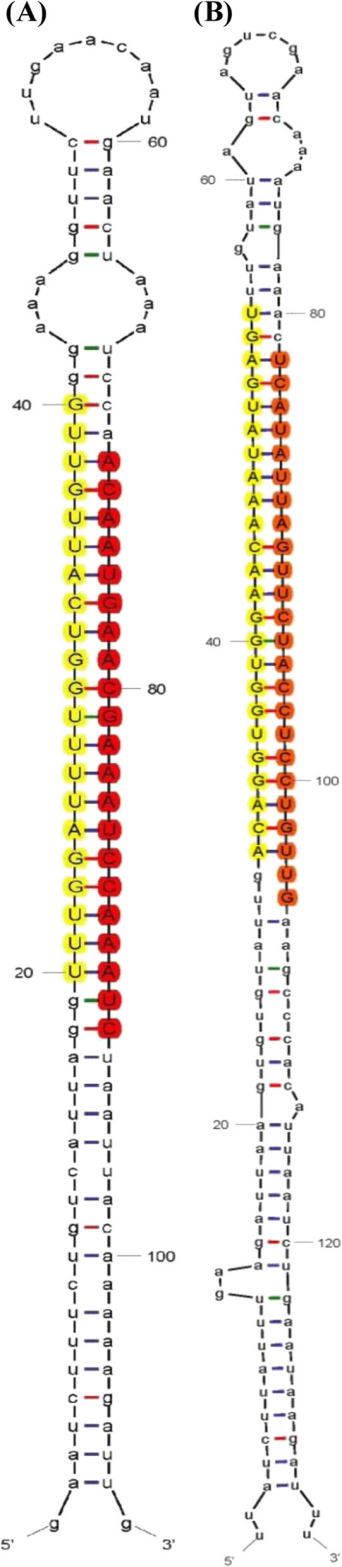
**Predicted secondary structures of novel miRNAs in *Brassica campestris*. (A)** bra-miRn9 **(B)** bra-miRn10-1.

To predict novel miRNAs in *Brassica campestris*, all the mappable small RNAs were blasted to the *Brassica campestris* genome sequence in *Brassica* database and plant known miRNAs in miRBase. The small RNAs that exactly map to the genome sequence but not the plant known miRNAs were classified as candidate novel miRNAs. To increase predictive accuracy, five criteria described in the materials and methods were mainly used to search for novel miRNAs. Novel miRNAs were discovered in pairs since the miRNA/miRNA* criterion was used. As a result, 25 pairs of novel miRNAs/miRNA* that belong to 23 miRNA families were identified in this study (Table 3). The bra-miRn1 had two sub-members (bra-miRn1-1 and bra-miRn1-2), as well as bra-miRn10 (bra-miRn10-1 and bra-miRn10-2). The lengths of mature miRNAs were distributed in the range of 21 nt to 24 nt. The MFE of these predicted pre-miRNAs ranged from -37 kcal/mol to -209.8 kcal/mol. The MFEI ranged from 0.9 to 2.1, with an average of 1.3, which is consistent with the characteristics of miRNA. Most of these novel miRNAs were expressed in the flower buds of the A line and B line, but the expression levels were very low. bra-miRn22-3p showed obviously high expression abundance in the flower buds of the A line and B line, which was considerably higher than expression levels of other miRNAs. The information about the numbers of reads and the sequence characteristics of all the identified miRNAs by using high-throughput sequencing are summarized in Additional file 2: Table S5. The hairpin structures for precursors of bra-miRn9 and bra-miRn10-1 are used as examples in Figure 2.

**Table 3:** **Novel miRNAs identified in the A line and B line of *Brassica campestris *** **by high-throughput sequencing**

**miR_name**	**miR_seq**	**LM**	**LP**	**CG%**	**dG**	**MFEI**	**FbA**	**FbB**
bra-miRn1-1-5p	CTATCGGTCTACTCGGTCAGC	21	153	50	-131.5	1.2	19	7
bra-miRn1-1-3p	TGACCGAGTAGACCGATAGTC	21	153	50	-131.5	1.2	87	222
bra-miRn1-2-5p	CTATCGGTCTACTCGGTCAGC	21	153	47	-152.2	1.3	19	7
bra-miRn1-2-3p	TGACCGAGTAGACCGATAGTC	21	153	47	-152.2	1.3	87	222
bra-miRn2-5p	CAACAGTCTCAGGATGGAAAA	21	134	35	-47.6	1	3	12
bra-miRn2-3p	TTTCATCTTAGAGAATGTTGTT	22	134	35	-47.6	1	57	153
bra-miRn3-5p	TACAAAGCTGAAGCTAATTATG	22	141	41	-70.2	0.9	18	56
bra-miRn3-3p	TAATCAGCTCCAGCTATGTACA	22	141	41	-70.2	0.9	145	175
bra-miRn4-5p	GAATGATACTTGGATATGATC	21	147	34.7	-80.3	1.5	14	6
bra-miRn4-3p	TTATATCCAAGTATCATTCCT	21	147	34.7	-80.3	1.5	15	34
bra-miRn5-5p	TTCTAAGCTTTACGGGAAACC	21	201	29	-101.4	1.7	10	11
bra-miRn5-3p	TTTCCCGTAAAGCTTAGAACC	21	201	29	-101.4	1.7	12	7
bra-miRn6-5p	GTCAATTGGTGATAGTAGTTC	21	84	36.7	-38.3	1.2	11	41
bra-miRn6-3p	TCTACTTTCACCAATTGGCCT	21	84	36.7	-38.3	1.2	4	54
bra-miRn7-5p	TTTTGCGTTTCAACTCGGTCC	21	139	38.8	-64	0.9	73	61
bra-miRn7-3p	GCTGAGTTGGAACACAAAATC	21	139	38.8	-64	0.9	19	8
bra-miRn8-5p	AGAGATGTCTGGCTTGCAACA	21	140	44.5	-74.3	1.1	1	3
bra-miRn8-3p	TTGCAAGCCAGACATTTCCTTT	22	140	44.5	-74.3	1.1	5	9
bra-miRn9-5p	TTTGGATTTTGGTCATTGTTG	21	107	32.1	-50.7	1.4	0	2
bra-miRn9-3p	ACAATGAACGAAATCCAAATC	21	107	32.1	-50.7	1.4	4	9
bra-miRn10-1-5p	ACAGGTGGTGGAACAAATATGAGT	24	128	31.8	-52.5	1.3	1	13
bra-miRn10-1-3p	TCATATTAGTTCTACCTCCTGCTG	24	128	31.8	-52.5	1.3	2	7
bra-miRn10-2-5p	ACAGGTGGTGGAACAAATATGAGT	24	130	31.6	-44.4	1	1	13
bra-miRn10-2-3p	TCATATTAGTTCTACCTCCTGCTG	24	130	31.6	-44.4	1	2	7
bra-miRn11-5p	TGAGTCTCTCACCAGTCTTTCAC	23	117	34.1	-59.3	1.4	2	2
bra-miRn11-3p	GAGAGACTCTGAAAGACTCACC	22	117	34.1	-59.3	1.4	8	5
bra-miRn12-5p	TGTAATTGCGGGGTTCTAAGC	21	204	29.1	-103.6	1.7	7	9
bra-miRn12-3p	TTAGAAACCTGCAATTATATA	21	204	29.1	-103.6	1.7	3	3
bra-miRn13-5p	ACTATGCAATTGTGAACAAAC	21	128	29.5	-56.4	1.3	2	3
bra-miRn13-3p	TTATTCACAACTGCATAATTC	21	128	29.5	-56.4	1.3	2	0
bra-miRn14-5p	GGGAGCCAGGGAAGAGGCAGT	21	165	41.7	-66	0.9	0	2
bra-miRn14-3p	TGCTTGTTCCCTGTCTCTCTC	21	165	41.7	-66	0.9	4	1
bra-miRn15-5p	ACCCGTCTCTTAATTTTTAAC	21	161	31.7	-59.4	1.1	19	32
bra-miRn15-3p	TAAAAGTTAAGAGACAAGTTA	21	161	31.7	-59.4	1.1	0	1
bra-miRn16-5p	ATAAAACGATTACACAGCTCGGTC	24	230	42.1	-209.8	2.1	1	1
bra-miRn16-3p	CGAGCTGTGTAATCGTTTTGTTA	23	230	42.1	-209.8	2.1	1	0
bra-miRn17-5p	TCTCGTTCTCTCGTTTCAGCT	21	114	39.6	-56.6	1	0	3
bra-miRn17-3p	CTGAAGCTAGTGAAAGAGAGA	21	114	39.6	-56.6	1	0	2
bra-miRn18-5p	TTGTTGACAAATACTTAGGCTC	22	154	33.5	-121.6	1.7	3	7
bra-miRn18-3p	GAGCCTAAGTATTTGTCAACAATG	24	154	33.5	-121.6	1.7	0	7
bra-miRn19-5p	TAAACAACACATATACTTTGC	21	132	37	-89.6	1.8	0	2
bra-miRn19-3p	AAACTATATGTGTTGCTTAGA	21	132	37	-89.6	1.8	1	0
bra-miRn20-5p	AAGAACTCGTCTCTTAACTTTTAA	24	177	30.7	-86.7	1.2	1	5
bra-miRn20-3p	AAACTAAGAGATGAATTCTTAC	22	177	30.7	-86.7	1.2	1	1
bra-miRn21-5p	NGCGGATATCTTAGGATGAGGT	22	144	28.7	-55.8	1.3	0	1
bra-miRn21-3p	TCATCGTAAGAGATCTGCATT	21	144	28.7	-55.8	1.3	0	1
bra-miRn22-5p	TGAGTTATCATTGGTCTTGTG	21	186	28.1	-94.6	1.8	0	1
bra-miRn22-3p	ACACAGGAACAATACTAACTCATT	24	186	28.1	-94.6	1.8	2664	4123
bra-miRn23-5p	CTTTGTCTATCGTTTGGAAAAG	22	101	37.4	-37	0.9	25	95
bra-miRn23-3p	TTTCCAAATGTAGACAAAGCT	21	101	37.4	-37	0.9	0	1

miR_name, miRNA name; miR_seq, miRNA sequence; LM, length of mature miRNA; LP, length of precursor miRNA; dG, minimum free energy; MFEI, minimum folding free energy index; FbA, flower buds of A line; FbB, flower buds of B line.

### Expression profiling of differentially expressed miRNAs in the flower buds of A line and B line

**Figure 3:**
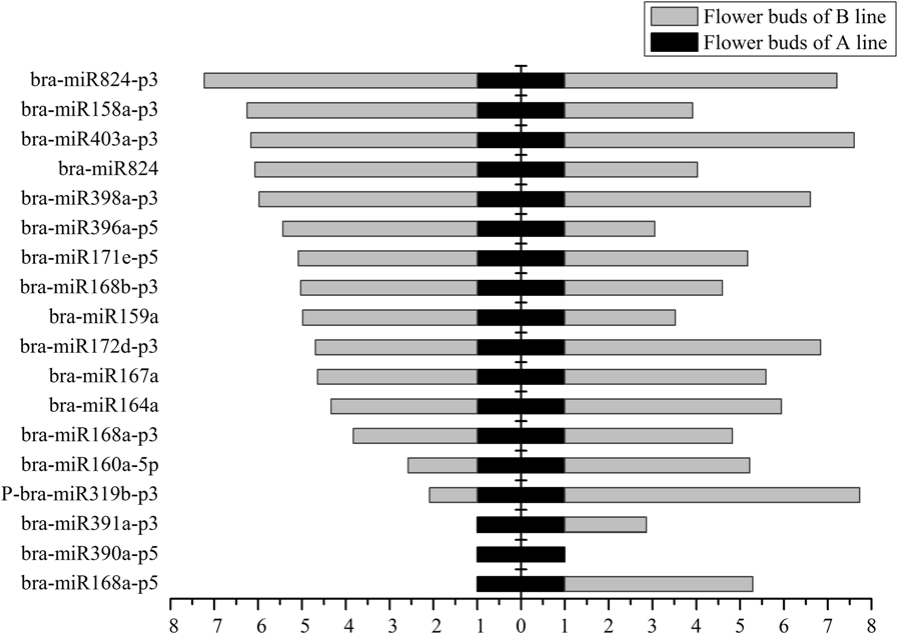
**Relative expression analysis of miRNAs in the flower buds of the A line and B line by high-throughput sequencing and qRT-PCR analysis.** Relative expression level was normalized to the expression level of 5.8SrRNA in qRT-PCR. All qRT-PCR reactions were prepared in triplicate for each sample. Left indicates the miRNA relative expression level generated from the high-throughput sequencing. Right indicates the miRNA relative expression level obtained by using qRT-PCR analysis.

**Figure 4:**
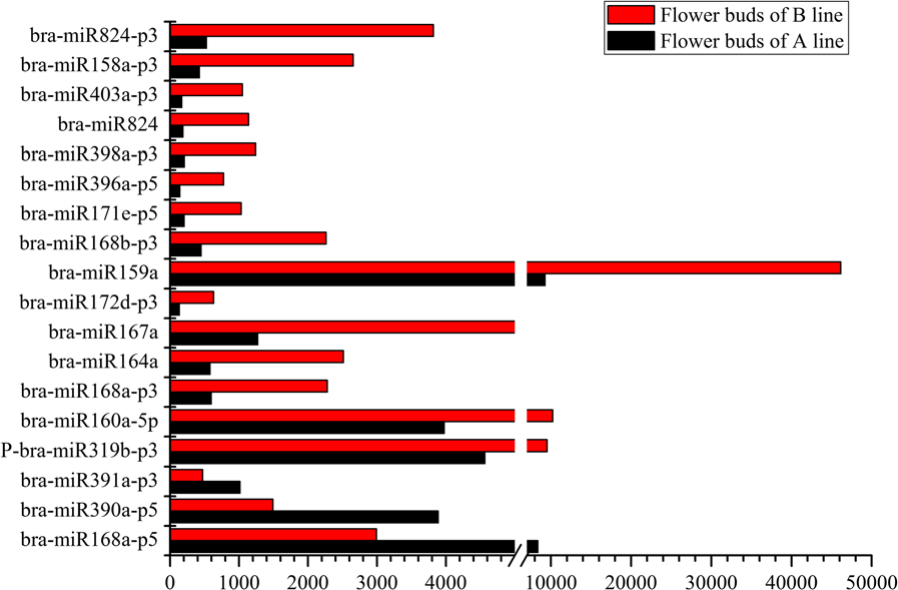
**Expression profiles of differentially expressed miRNAs in flower buds of the A line and B line by high-throughput sequencing.** The *Y* axis represents the number of reads of miRNAs detected in small RNA libraries from flower buds of A line (black bars) and B line (red bars) by using high-throughput sequencing.

Throughout all the identified conserved and novel miRNAs, 18 differentially expressed miRNAs with more than two-fold relative change between the flower buds of A line and B line were identified in high-throughput sequencing (Figure 3). The relative expression level was calculated based on the normalized number of sequence reads of these miRNAs in small RNA libraries from flower buds of A line and B line. Among the 18 differentially expressed miRNAs, 15 miRNAs were up-regulated in flower buds of the B line. The remaining three miRNAs (bra-miR391a-p3, bra-miR390a-p5, and bra-miR168a-p5) showed higher expression levels in the flower buds of A line than in the B line (Figure 3). For all 15 miRNAs enriched in flower buds of the B line, bra-miR824-p3 showed the highest relative expression level (7.23-fold). For the three miRNAs enriched in flower buds of the A line, the differential expression of bra-miR168a-p5 was the most obvious (2.80-fold). qRT-PCR was conducted to verify the expression profile of the 18 differentially expressed miRNAs in deep sequencing. The results of qRT-PCR largely agreed with the deep sequencing results (Figure 3). In qRT-PCR, two more miRNAs (bra-miR391a-p3 and bra-miR168a-p5) were found to be up-regulated in the B line, which were enriched in the A line based on deep sequencing analysis. bra-miR390a-p5 was also up-regulated in the A line based on qRT-PCR analysis. In addition, expression profiles of these 18 miRNAs were presented in terms of the number of reads in flower buds of the A line and B line libraries (Figure 4). The number of normalized reads of bra-miR159a was very high, followed by bra-miR160a-5p, P-bra-miR319b-p3, and bra-miR168a-p5. P-bra-miR319b-p3 was predicted to be the 3′ arm miRNA of bra-miR319b. For all the identified miRNAs in this study, 5′ arm and 3′ arm miRNAs, namely, miRNA and miRNA*, were both detected, except for P-bra-miR319b-p3. Therefore, a “P” was added to bra-miR319b-p3, which denotes “predicted”. The two members of bra-miR168 family, namely, bra-miR168a-p3 and bra-miR168b-p3, were both up-regulated in flower buds of the B line. bra-miR824-p3 and bra-miR824 were consistently up-regulated in flower buds of the B line, which indicates that different members can have similar expression patterns.

### Identification of miRNA target genes in *Brassica campestris* by using degradome analysis

**Figure 5:**
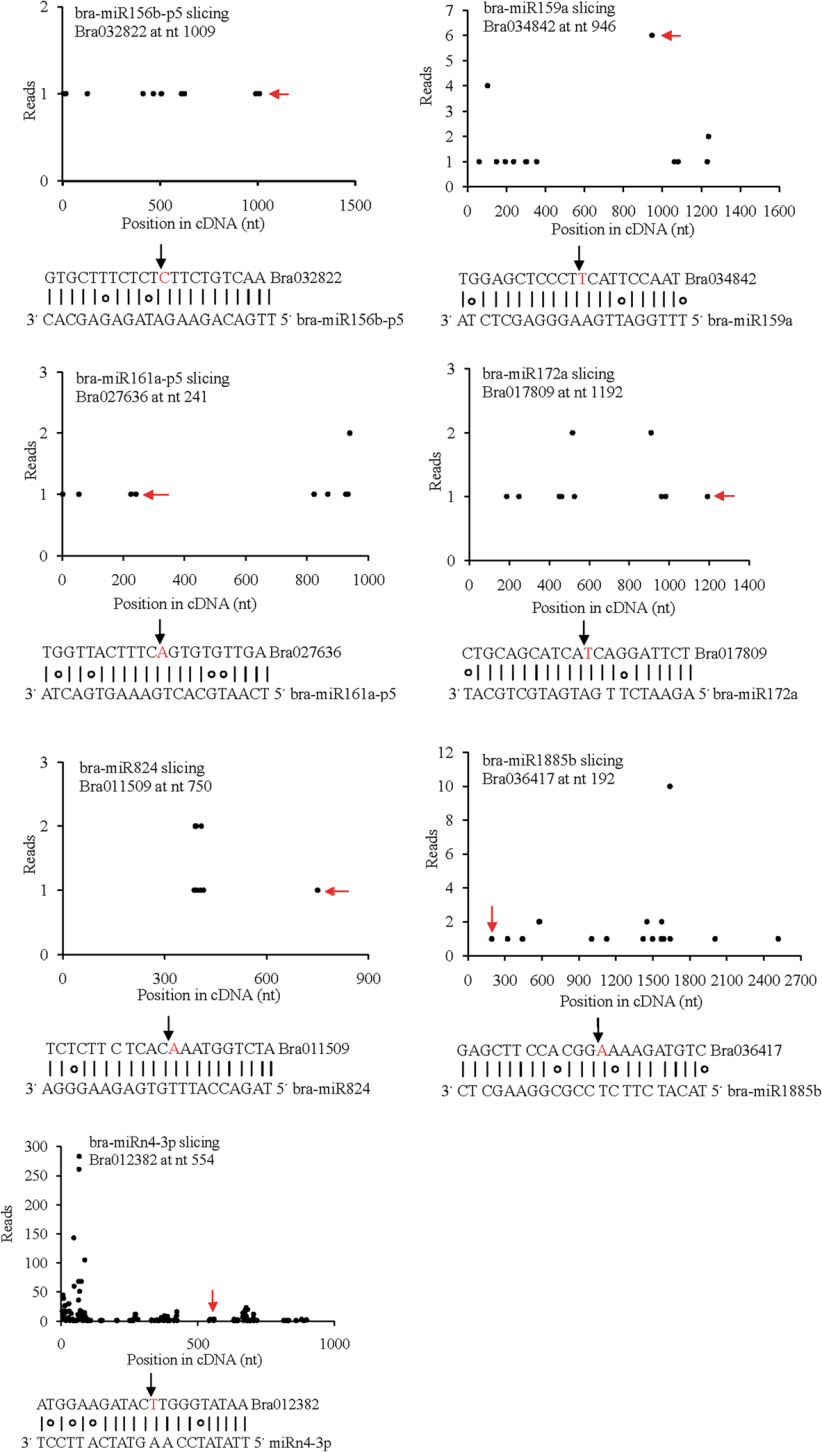
**Target plots (t-plots) of miRNAs targets confirmed by using degradome sequencing.** A degradome cDNA library was constructed from the inflorescences of the B line and subjected to Illumina sequencing. A t-plot (top) and the corresponding miRNA:mRNA alignment (bottom) are shown for each of the seven target transcripts. In t-plots, the red arrows indicate the miRNA-directed cleaved transcript. The *X* axis indicates the nucleotide position in target cDNA. The *Y* axis indicates the number of reads of cleaved transcripts detected in the degradome cDNA library. The solid lines and dots in miRNA:mRNA alignments indicate matched and mismatched base pairs, respectively, and the red arrows and letters indicate the cleavage sites.

Target identification of the miRNAs is important to further understand the potential regulatory role and biological function of a miRNA. In this study, degradome sequencing was used to search for the target genes of identified miRNAs in *Brassica campestris*. A total of 15 targets were predicted to be cleaved by seven miRNAs (Table 4). The seven miRNAs were bra-miR156, bra-miR159, bra-miR161, bra-miR172, bra-miR824, bra-miR1885, and bra-miRn4. bra-miR156 was identified in this study and was found to be highly conserved in plant species. Three targets that encode squamosa promoter binding protein (SBP) transcription factors were identified for bra-miR156. bra-miR159 and bra-miR172 were highly conserved miRNAs. They respectively targeted three and two transcription factors that belong to *MYB* domain protein family and *AP2*-like factor, respectively. bra-miR161 and bra-miR824 were both Cruciferae-specific miRNAs. bra-miR161 was identified in the present deep sequencing analysis and was found to target two pentatricopeptide repeat (PPR)-containing protein family genes. bra-miR824 is a known miRNA, which was predicted to cleave an AGAMOUS-like transcription factor. bra-miR1885 is a known miRNA that is only reported in *Brassica campestris* and might be specific only to *Brassica*. bra-miR1885 was predicted to target two genes encoding disease resistance protein (TIR-NBS-LRR class). bra-miRn4 is a novel miRNA that was predicted to cleave two genes that encode unknown proteins. Most of the identified targets are generally homologous to target genes found in *Arabidopsis* (Table 4). Unfortunately, for most miRNAs identified through deep sequencing, including conserved miRNAs and novel miRNAs, their target genes could not be detected in the present degradome analysis. In the above analysis, 15 target genes were identified for seven miRNA families. Among the 15 targets, nine were transcription factors. For each miRNA family, one target was chosen and a t-plot was constructed (Figure 5). In t-plots, the cleavage site for each miRNA:mRNA alignment is shown. The t-plots for the remaining eight targets are illustrated in Additional file 3: Figure S1.

**Table 4:** **Target genes identified by degradome sequencing in *Brassica campestris***

**miRNA family**	**Target gene**	**Target description**	**Homologous to *Arabidopsis***	**Cleavage site**	**Reads**	**Category**	**Conserved in *A. thaliana***
bra-miR156	Bra032822	Squamosa promoter-binding protein-like 11, transcription factor	AT1G27360 (SPL11)	1009	1	4	Y
	Bra010949	Squamosa promoter-binding protein-like 10, transcription factor	AT1G27370 (SPL10)	1006	1	4	Y
	Bra030041	Squamosa promoter-binding protein-like 10, transcription factor	AT1G27370 (SPL10)	1030	1	4	Y
bra-miR159	Bra034842	MYB domain protein 65; DNA binding/transcription factor	AT3G11440 (ATMYB65)	946	6	2	Y
	Bra002042	MYB domain protein 65; DNA binding/transcription factor	AT3G11440 (ATMYB65)	934	6	0	Y
	Bra035547	MYB domain protein 120; DNA binding/transcription factor	AT5G55020 (ATMYB120)	1120	1	4	Y
bra-miR161	Bra027636	PPR repeat-containing protein	AT1G64580	241	1	4	
	Bra028267	PPR repeat-containing protein	AT1G12300	982	1	4	
bra-miR172	Bra017809	AP2 (APETALA 2)-like factor; transcription factor	AT4G36920 (AP2)	1192	1	4	Y
	Bra011741	AP2 (APETALA 2)-like factor; transcription factor	AT4G36920 (AP2)	1192	1	4	Y
bra-miR824	Bra011509	AGL16 (AGAMOUS-like 16); transcription fctor	AT3G57230 (AGL16)	750	1	4	Y
bra-miR1885	Bra036417	Disease resistance protein (TIR-NBS-LRR class), putative	AT2G14080	192	1	4	
	Bra038872	Disease resistance protein (TIR-NBS-LRR class), putative	AT5G11250	198	1	4	
bra-miRn4	Bra012382	Unknown protein	AT2G27670	554	3	3	
	Bra012383	Unknown protein	AT1G23560	1439	3	3	

## Discussion

### Characteristics of conserved and non-conserved miRNAs in plants

In plants, many miRNAs seems to be universally expressed among diverse angiosperms, such as miR156, miR159, miR160, miR162, miR171, miR172 and so on. Among them, a small number of miRNAs have also been detected in bryophyte, lycopod, gymnosperm, such as miR156, miR319 [25]. However, there are a large number of miRNAs which are just present in a few species, even in only one species. For example, miR415, miR416, miR417, and miR418 have only been detected in *Arabidopsis* and *Oryza sativa*[26,27]. miR1885 and miR5718 have just been identified in *Brassica campestris*[28,29]. Considering that some miRNAs are widespread, while others distribute in limited plant species, miRNAs are classified into two categories, ‘conserved’ miRNAs and ‘non-conserved’ miRNAs. In previous reports, the term ‘conserved’ miRNAs are mainly used when the miRNAs are present throughout at least one major ancient clade of land plants, for example angiosperms. ‘Non-conserved’ miRNAs are defined as those with a limited phylogenetic distribution and characterized by primarily being single-copy genes [25]. In present years, with the development of high-throughput sequencing, a high proportion of non-conserved miRNAs have been identified in many species, such as larch [30], *Populus euphratica*[31], rice [32], maize [33], and soybean [34]. In our study, 15 conserved miRNA families, which had not been previously reported in *Brassica campestris* but reported in other plant species, were identified*.* Meanwhile, 25 pairs of novel miRNA/miRNA* were identified according to strict miRNA/miRNA* identification criteria. They are likely to be *Brassica campestris*-specific miRNAs, of course which are classified into non-conserved miRNAs.

Previous reports have indicated that non-conserved miRNAs are often species specific, weakly expressed, and encoded by single loci [35], while highly conserved miRNAs are widespread, highly expressed, and most of them have more than one family member [36].Our results were just in accord with the previous results. In our study, the numbers of reads of the 25 pairs of novel miRNA/miRNA* were extremely low, except for bra-miRn22-3p. Most of their read numbers were less than 100. However, the read numbers of the conserved miRNAs were very high. Most of their read numbers were more than 1000. bra-miR159 had the highest number of reads, which was close to 45000. A similar phenomenon was also observed in *Arabidopsis lyrata*[35,37]. In addition, in our study, except for bra-miRn1 and bra-miRn10 families, only one member was identified for the rest of 21 novel miRNA families, while most of the conserved miRNA families have more than two family members. Our results indicates that compared with conserved miRNAs, most of the novel miRNAs were encoded by a single locus., which was consistent with the previous study [35]. In general, all of the previous studies and our results verify that conserved and non-conserved miRNAs present nearly opposite characters. As for why they show so different characters, more research on evolution and functional diversification of *MIRNA* genes will be helpful to elucidate it.

### Diverse miRNAs are present in pollen and they are possibly involved in pollen development

Previous studies have demonstrated that many miRNAs exist in *Arabidopsis* mature pollen. In 2009, Grant-Downton et al. detected 33 known miRNAs in *Arabidopsis* pollen by using 454 sequencing technology [24]. In the same year, Chambers and Shuai verified that many miRNAs are expressed in the pollen and inflorescences of *Arabidopsis* using miRNA array [23]. Many studies have also been conducted in rice. In 2011, Wei et al. used deep sequencing technology to analyze the composition and expression patterns of miRNAs in developing pollen of rice, including uninucleate microspores, bicellular pollen and tricellular pollen, as well as sporophytic tissues. A total of 292 known miRNAs and 75 novel miRNAs were detected. Among the 292 known miRNAs, 103 were enriched in developing pollen and more than half of the novel miRNAs displayed pollen- or stage-specific expression. These pollen- or stage-specific miRNAs might function during pollen development process [22]. In our study, we identified 24 known miRNAs, 54 conserved miRNAs, and 25 pairs of novel miRNA/miRNA* in flower buds of the A line and B line plants. Among all these miRNAs, 18 differentially expressed miRNAs with more than two-fold relative change between flower buds of A line and B line were identified. Moreover, most of the 18 differentially expressed miRNAs were up-regulated in flower buds of B line. We speculate that these miRNAs might be involved in pollen development process. Previous studies and the present study demonstrate that diverse miRNAs exist in plant pollen and they might have potential regulatory roles in pollen development.

In 2009, Grant-Downton et al. demonstrated that several mainly sRNA pathway genes, including *AGO* family members, *DCL1-4*, *HASTY*, *SERRATE*, *HEN1*, and *RDR* family members, were expressed in unicellular microspores, bicellular pollen, tricellualr pollen, and mature pollen grains in *Arabidopsis* by RT-PCR analysis. At the same time, they succeeded in amplifying the pri-miRNAs, pre-miRNAs, and mature miRNAs from mature pollen cDNA, which verified that miRNA synthesis in pollen was going on as usual [38]. Their results further verify that miRNAs are not only present in pollen, but also may participate in the complex regulatory network of pollen development.

### The application of degradome analysis have massively accelerated the research on the interactions of miRNAs and their target genes

Identification of target genes is the first and essential step to understand the regulatory roles of miRNAs. In plants, most miRNAs can perfectly or almost perfectly bind to their target mRNAs. Thus, bioinformatics prediction is a common approach for predicting miRNA target genes. In recent years, many computational tools have been developed for predicting plant miRNA targets, such as psRNATarget [39], Target-align [40], TAPIR [41], and PMRD [42]. 5′-modified RACE is frequently used to demonstrate miRNA targets. This method is easy to operate, and the results are reliable. However, 5′-modified RACE is based on the premise that the candidate target is already predicted and its mRNA sequence is known for primer design. 5′-modified RACE is not efficient for identifying a large number of candidate target genes. Fortunately, in recent years, degradome sequencing analysis [43], a new method for massively identifying target genes, is increasingly being developed for such applications. The application of this new method greatly accelerates the detection of miRNA targets.

In recent years, degradome sequencing has been used for large-scale target identification in many species, such as *Populus euphratica*[31], soybean [34], cucumber [44], and *Brassica juncea*[45]. In the present study, degradome sequencing was performed and finally a large number of candidate target genes were detected. In order to confirm the reliable candidate targets, two criteria described in the materials and methods were mainly used. Finally, a total of 15 targets were chosen and predicted to be cleaved by seven miRNA families. Nine of the 15 targets were transcription factors. They were squamosa promoter binding (SBP) transcription factors (Bra032822, Bra010949, Bra030041), MYB transcription factors (Bra034842, Bra002042, Bra035547), AP2-like transcription factors (Bra017809, Bra011741), AGAMOUS-like transcription factors (Bra011509), which were targeted by miR156, miR159, miR172, miR824 families, respectively. These target genes have been reported playing an important role in plant growth and development. miR172, which targets AP2-like transcription factors, has been implicated in the regulation of flowering time and floral organ identity in maize and *Arabidopsis*[16,17]. In 2009, Wu et al. indicated that miR156 and miR172 regulated the development transition from juvenile to adult [46]. In the next year, Xing et al. concluded that fully fertile *Arabidopsis* flowers required the action of multiple miR156/7-targeted *SPL* genes in concert with *SPL8*, otherwise semi-sterile or fully sterile would emerge [47]. The results suggested that miR156/7 and their targets, namely SBP transcription factors, might participate in gametophyte development. miR159 and its target genes, namely MYB-like genes, were proved to inhibit growth and promote programmed cell death in *Arabidopsis*[48]. In addition to targeting transcription factors, the remaining 6 target genes were also shown to be involved in other biological processes. For example, miR1885 and its two targets were involved in disease resistance [29]. miR161 and bra-miRn4 both had two candidate target genes and their functions in plants were unknown yet. In summary, degradome analysis has greatly accelerated the identification of miRNA targets. Meanwhile, it speeds up the research on miRNA/target interactions.

## Conclusion

In this study, a large number of miRNAs were identified in *Brassica campestris* ssp. *chinensis*, including 8 novel miRNAs on the other arm of known pre-miRNAs, 54 conserved miRNA families, 8 new miRNA members that belong to 8 known miRNA families, and 25 pairs of novel miRNA/miRNA*. Meanwhile, by analyzing the sequencing reads of miRNAs, we found that there were18 miRNAs differentially expressed between the flower buds of A line and B line, and 15 of them were up-regulated in flower buds of B line. This result was validated by using qRT-PCR analysis. By using degradome sequencing, a total of 15 targets were identified for 7 miRNA families, which reveal interaction between miRNAs and targets. In a word, the identification of plenty of miRNAs has greatly enriched the existing miRNA group in *Brassica campestris*. More importantly, the present study might provide valuable clues for exploring miRNA-mediated regulatory networks during pollen development.

## Methods

### Plant materials, sample collection, and total RNA extraction

A genic male sterile system in Chinese cabbage-pak-choi (*Brassica campestris* ssp. *chinensis*, syn. *Brassica rapa* ssp. *chinensis*), named ‘*Bcajh97-01A/B*’ was used in this study. ‘*Bcajh97-01A*’ is a male sterile line that lacks mature pollen, and its male sterility is controlled by a pair of nuclear recessive genes. ‘*Bcajh97-01B*’ is the fertile line that generates normal mature pollen and is the maintainer line of ‘*Bcajh97-01A*’. The ‘*Bcajh97-01A/B*’ sister line system has been developed through continuous backcrossing within the population for several generations [49]. The progenies of ‘*Bcajh97-01A/B*’ line regularly segregate into sterile and fertile types during reproduction at a ratio of 1:1. The characteristics of male sterility can be steadily maintained.

All plant materials were grown in the experimental farm of Zhejiang University. In this study, three kinds of samples were harvested at the flowering stage from ‘*Bajh97-01A/B*’ plants, which were the mixture of flower buds from ‘*Bcajh97-01A*’ and ‘*Bcajh97-01B*’ and the inflorescences from ‘*Bcajh97-01B*’ , respectively. Each kind of sample was collected from 10 different plants and subsequently mixed, flash frozen in liquid nitrogen, and stored at -80°C until total RNA isolation. Total RNAs of the three kinds of samples were extracted by using mirVana kit (Ambion, USA) according to the manufacturer’s instructions.

### Small RNA library construction and sequencing

Small RNA library construction was conducted by using Illumina TruSeq Small RNA Preparation Kit following the manufacturer’s instructions (LC Sciences, Hangzhou, China). The general process is as follows: first, the total RNA was ligated to RNA 3′ and RNA 5′ adapters. Second, reverse transcription followed by PCR was performed to create cDNA constructs based on the small RNAs ligated with 3′ and 5′ adapters. Third, small cDNA fractions that range from 22 nt to 30 nt in length were isolated by using 6% denaturing polyacrylamide gel electrophoresis. Fourth, cDNA construct was purified, and the library was validated.

The purified cDNA library was used for cluster generation on Illumina’s Cluster Station and subsequently sequenced on Illumina GAIIx (Illumina, Inc., Santa Clara, CA) following the manufacturer’s instruction on running the instrument. Raw sequencing reads were obtained by using related Illumina’s analysis software. The ACGT101-miR program (version 4.2; LC Sciences) was used for standard sequencing data analysis.

### Identification of conserved and novel miRNAs

After the raw sequence reads were extracted, adapter sequences, impurities, and sequences beyond 15 nt to 30 nt were filtered. The remaining sequences that range from 15 nt to 30 nt in length were used for miRNA prediction by using the ACGT101-miR program (version 4.2; LC Sciences). First, the sequences were blasted to the RFam database (RFam: rRNA, tRNA, snRNA, snoRNA, and other non-coding RNAs), repeat sequences, and mRNAs. Matched sequences were discarded. The sequences were then compared with the *Brassica campestris* genome sequences downloaded from the *Brassica* database (http://brassicadb.org/brad/). The unmatched sequences were filtered. Finally, the remaining sequences were mapped to all known plant miRNAs sequences to identify the conserved miRNAs in *Brassica campestris* from the miRBase database (version 19.0, http://www.mirbase.org/). Matched sequences with no more than three mismatches were considered as candidate conserved miRNAs*.* At the same time, the unmatched sequences were reserved as candidate novel miRNAs. To identify conserved or novel miRNAs in *Brassica campestris*, novel and conserved candidate miRNAs sequences were blasted against *Brassica campestris* genome sequences, and their flanking sequences in the genome were used to predict their secondary structures by using the mfold Web server (http://mfold.rna.albany.edu/?q=mfold/download-mfold) [50]. A potential miRNA precursor must be a non-coding sequence and must meet certain criteria. The first and the most important criterion is the miRNA/miRNA* criterion. Both a candidate miRNA and its corresponding reverse sequence, namely, the candidate miRNA* sequence, must be detected in the present high-throughput sequencing. Second, the candidate miRNA and miRNA* sequences must be found on the stem, and the number of mismatched bases between them must be less than four (four continuous mismatches are also not allowed). Third, within the miRNA/miRNA* duplex, the number of asymmetric bulges must be one or fewer, and the number of bases in the asymmetric bulges must fewer than two. Fourth, the miRNA and miRNA* should be located in opposite stem-arms and form a duplex with two nucleotide 3′ overhangs [51]. Fifth, the potential miRNA precursor must have higher negative minimal folding energy (MFE) and minimal folding energy indexes (MFEI), with the MFEI > 0.8, to distinguish from other small RNAs [52]. After the above strict screening, conserved and novel miRNAs are identified in *Brassica campestris*.

### Degradome library construction, data analysis, and target identification

A degradome library was constructed from the inflorescences of the fertile line (*Bcajh97-01B*) based on the method described by German et al. [43] and Addo-Quaye [53]. Briefly, poly(A)-enriched RNA was ligated to a 5′-RNA adapter with 3′ a *EcoP15* I recognition site. Reverse transcription was performed to generate first-strand cDNA, followed by PCR amplification and *EcoP15* I digestion. After digestion with *EcoP15* I, a PAGE-gel was used to purify the *EcoP15* I-cleaved fragments. The gel-purified products were ligated to a 3′-double-strand DNA adapter, followed by PAGE-gel purification to obtain the ligated products. PCR amplification was performed, and PAGE-gel was used for the third time to purify the corresponding gel bands containing the final products. Finally, the purified cDNA library was ready for deep sequencing (LC Sciences, Hangzhou, China).

The purified cDNA library was first used for cluster generation on Illumina’s Cluster Station and then sequenced on Illumina GAIIx. Raw sequencing reads were obtained by using Illumina’s Pipeline v1.5 software following sequencing image analysis by Pipeline Firecrest Module and base-calling by using Pipeline Bustard Module (LC Sciences, Hangzhou, China). A public software package, CleaveLand 3.0, was used for analyzing sequencing data [53,54].

By degradome sequencing, a great many genes may be predicted as potential target genes. In this study, two criteria were mainly used to choose reliable genes as candidate targets. First, the cleavage site must be the 9th or 10th nucleotide of the target mRNA in the miRNA/target binding region. Second, the candidate targets must be homologous to corresponding *A. thaliana* targets [55]. The candidate target genes that meet the above criteria would be identified as targets.

### Quantitative real-time PCR

Total RNA was extracted from the flower buds of the sterile line and the fertile line by using the mirVana kit (Ambion, USA). According to the procedures provided by a miRNA cDNA synthesis kit (TaKaRa, Japan), 1 μg of total RNA was polyadenylated with ATP by poly(A) polymerase. The poly(A)-tailed total RNA was reverse-transcribed by PrimeScript® RTase by using a universal adapter primer (containing oligo-dT). qRT-PCR analysis was carried out by using SYBR® Premix Ex TaqTM II (Perfect Real Time) (TaKaRa, Japan) on a Bio-Rad CFX96 machine. All reactions were performed in triplicate for each sample, and 5.8S rRNA was used as the internal control gene. Relative expression levels of miRNAs were quantified by using the 2^-ΔΔCt^ method [56]. The primers used for qRT-PCR are listed in Additional file 1: Table S1.

## Competing interests

The authors declare that they have no competing interests.

## Authors’ contributions

JXJ and JSC designed the study. JXJ performed the experiments, analyzed the data, and drafted the manuscript. YL and MLL assisted with bioinformatic analysis and aided in writing the manuscript. ZMM aided in performing the experiments. All authors carefully checked and approved this version of the manuscript.

## Supplementary Material

Additional file 1: Table S1 Primer sequences used for quantitative RT-PCR analyses. **Table S2.** Identification of known miRNAs. **Table S3.** Identification of new conserved miRNAs in *Brassica campestris.***Table S4.** Identification of new miRNA family members by high-throughput sequencing. Click here for file

Additional file 2: Table S5 Information about the numbers of reads and the sequence characteristics of all the identified miRNAs by using high-throughput sequencing. Click here for file

Additional file 3: Figure S1 Target plots (t-plots) of miRNAs targets confirmed by using degradome sequencing in *Brassica campestris.* Click here for file
