# Supplementary material for: Identification of novel and conserved miRNAs involved in pollen development in Brassica campestris ssp. chinensis by high-throughput sequencing and degradome analysis
Source: BMC Genomics. 2014 Feb 21;15:146. doi: 10.1186/1471-2164-15-146 (PMC3936892; doi:10.1186/1471-2164-15-146)
Supplement: Additional file 1: Table S1 — Primer sequences used for quantitative RT-PCR analyses. Table S2. Identification of known miRNAs. Table S3. Identification of new conserved miRNAs in Brassica campestris.Table S4. Identification of new miRNA family members by high-throughput sequencing. [file 1471-2164-15-146-S1.pdf]

Table S1 Primer sequences used for quantitative RT-PCR analyses

miRNA	Primer sequence
bra-miR168a-p5 (bra-miR168a-1-p5, bra-miR168a-2-p5)	TCGCTTGGTGCAGGTCGGGA
bra-miR390a-p5	AAGCTCAGGAGGGATAGCGCC
bra-miR391a-p3	CGACGGTATCTCTCCTACGTAGC
P-bra-miR319b-p3	TTGGACTGAAGGGAGCTCCTT
bra-miR160a-5p	TGCCTGGCTCCCTGTATGCCA
bra-miR319a-p3	TTGGACTGAAGGGAGCTCCCT
bra-miR168a-p3 (bra-miR168a-1-p3,bra-miR168a-2-p3)	CCCGCCTTGCATCAACTGAAT
bra-miR164a,bra-miR164c-p5,bra-miR164d-p5	TGGAGAAGCAGGGCACGTGCA
bra-miR167a,bra-miR167b,bra-miR167d	TGAAGCTGCCAGCATGATCTA
bra-miR172d-p3	AGAATCTTGATGATGCTGCAG
bra-miR159a	TTTGGATTGAAGGGAGCTCTA
bra-miR168b-p3	CCCGCCTTGTATCAAGTGAAT
bra-miR171e-p5	TATTGGCCTGGTTCACTCAGA
bra-miR396a-p5	GTTCCACAGCTTTCTTGAACTT
bra-miR398a-p3	TGTGTTCTCAGGTCACCCCTG
bra-miR824	TAGACCATTTGTGAGAAGGGA
bra-miR403a-p3	TTAGATTCACGCACAAACTCG
bra-miR158a-p3	GCCTTTCCAAATGTAGACAAAGCA
bra-miR824-p3	CCTTCTCATCGATGGTCTAGA
bra-5.8S rRNA forward primer	CGATGAAGAACGTAGCGAAATG
bra-5.8S rRNA reverse primer	CTCGATGGTTCACGGGATTC

Table S2 Identification of known miRNAs

miR_name	Flower buds of A line	Flower buds of B line	miR_name	Flower buds of A line	Flower buds of B line
bra-miR159a	9,115	46,883	bra-miR172a	578	738
bra-miR160a-3p	48	293	bra-miR824	185	1,159
bra-miR160a-5p	3,913	10,405	bra-miR1140	816	429
bra-miR164a	570	2,555	bra-miR1885b	23,587	14,321
bra-miR167a	1,251	6,005	bra-miR2111b-3p	224	82
bra-miR167b	1,251	6,005	bra-miR5714	111	89
bra-miR167d	1,251	6,005	bra-miR5715	0	2
bra-miR171a	44	39	bra-miR5716	0	5
bra-miR171b	44	39	bra-miR5717	7	1
bra-miR171c	44	39	bra-miR5718	2,519	4,410
bra-miR171d	44	39	bra-miR5724	23,385	17,960
bra-miR171e	11,808	6,709	bra-miR5654a	81	536

Table S3 Identification of new conserved miRNAs in *Brassica campestris*

miR_name	miR_seq	len	Flower buds of A line	Flower buds of B line
bra-miR156a-p5	TGACAGAAGAGAGTGAGCAC	20	655	454
bra-miR156a-p3	GCTTACTCTCTCTCTGTCACC	21	968	1223
bra-miR156b-p5	TTGACAGAAGATAGAGAGCAC	21	903	582
bra-miR156b-p3	GCTCTCTATACTTCTGTCACC	21	6	2
bra-miR156c-p5	TGACAGAAGAGAGTGAGCAC	20	655	454
bra-miR156c-p3	GCTCACTCTCTATCCGTCACC	21	21	25
bra-miR156d-p5	TGACAGAAGAGAGTGAGCAC	20	655	454
bra-miR156d-p3	GCTCACTGCTCTATCTGTCAGA	22	0	14
bra-miR156e-p5	TGACAGAAGAGAGTGAGCAC	20	655	454
bra-miR156e-p3	GCTCACTCTCTATCTGTCACC	21	12	11
bra-miR156f-p5	TGACAGAAGAGAGTGAGCAC	20	655	454
bra-miR156f-p3	TGCTCACCTCTCTTTCTGTCAGT	23	44	220
bra-miR156g-p5	TGACAGAAGAGAGTGAGCAC	20	655	454
bra-miR156g-p3	TGCTCACTGCTCTTTCTGTCAGA	23	41	289
bra-miR156h-p5	TGACAGAAGAGAGTGAGCAC	20	655	454
bra-miR156h-p3	GCTCACTGCTCTATCTGTCAGG	22	0	5
bra-miR158a-5p	CTTTGTCTATCGTTTGGAAAAG	22	25	95
bra-miR158a-3p	TTTCCAAATGTAGACAAAGCA	21	418	2695
bra-miR161a-p5	TCAATGCACTGAAAGTGACTA	21	41	139
bra-miR161a-p3	GTCACTTTCAATGCGTTGATC	21	3	12
bra-miR162a-p5	GGAGGCAGCGGTTCATCGATC	21	43	16
bra-miR162a-p3	TCGATAAACCTCTGCATCCAG	21	225	485
bra-miR168a-1-p5	TCGCTTGGTGCAGGTCGGGAC	21	8250	3043
bra-miR168a-1-p3	CCCGCCTTGCATCAACTGAAT	21	587	2317
bra-miR168a-2-p5	TCGCTTGGTGCAGGTCGGGAC	21	8250	3043
bra-miR168a-2-p3	CCCGCCTTGCATCAACTGAAT	21	587	2317
bra-miR168b-p5	TCGCTTGGTGCAGGTCGGGAA	21	2802	5032
bra-miR168b-p3	CCCGCCTTGTATCAAGTGAAT	21	450	2263
bra-miR319a-p5	AGAGCTTCCTTGAGTCCATTC	21	113	63
bra-miR319a-p3	TTGGACTGAAGGGAGCTCCCT	21	4438	13370
bra-miR390a-p5	AAGCTCAGGAGGGATAGCGCC	21	3826	1518
bra-miR390a-p3	CGCTGTCCATCCTGAGTTTCA	21	153	270
bra-miR391a-p5	TTCGCAGGAGAGATAGCGCCA	21	62	241
bra-miR391a-p3	ACGGTATCTCTCCTACGTAGC	21	999	479
bra-miR395a-1-p5	GTTCCTCTGAGCACTTCATTG	21	3	12
bra-miR395a-1-p3	CTGAAGTGTTTGGGGGAACTC	21	776	633
bra-miR395a-2-p5	GTTCCTCTGAGCACTTCATTG	21	3	12
bra-miR395a-2-p3	CTGAAGTGTTTGGGGGAACTC	21	776	633
bra-miR395a-3-p5	GTTCCTCTGAGCACTTCATTG	21	3	12
bra-miR395a-3-p3	CTGAAGTGTTTGGGGGAACTC	21	776	633

bra-miR395a-4-p5	GTTCCTCTGAGCACTTCATTG	21	3	12
bra-miR395a-4-p3	CTGAAGTGTTTGGGGGAACTC	21	776	633
bra-miR396a-p5	TTCCACAGCTTTCTTGAACTT	21	140	788
bra-miR396a-p3	GCTCAAGAAAGCTGTGGGAAA	21	100	534
bra-miR398a-5p	GGGTCGACATGAGAACACATG	21	75	215
bra-miR398a-3p	TGTGTTCTCAGGTCACCCCTG	21	207	1242
bra-miR400a-p5	TATGAGAGTATTATAAGTCAC	21	104	75
bra-miR400a-p3	GACTTATAATGATCTCATGAA	21	1	5
bra-miR403a-p5	TGTTTTGTGCGTGAATCTAATT	22	5	48
bra-miR403a-p3	TTAGATTCACGCACAAACTCG	21	168	1067
bra-miR860a-p5	ATGTAGTCCAATCTATTGAAG	21	10	21
bra-miR860a-p3	TCAATACATTGGACTACATAT	21	18	50
bra-miR6032a-p5	AACATGGAGCATCAACAGATC	21	62	21
bra-miR6032a-p3	TCTGCTGGTCGTTCCATGTTAA	22	89	303

Table S4 Identification of new miRNA family members by using high-throughput sequencing

miR_name	miR_seq	len	Flower buds of A line	Flower buds of B line
bra-miR164b-1-p5	TGGAGAAGCAGGGCACGTGCG	21	8	60
bra-miR164b-1-p3	CACGTGTTCTACTACTCCAAC	21	35	24
bra-miR164b-2-p5	TGGAGAAGCAGGGCACGTGCG	21	8	60
bra-miR164b-2-p3	CACGTGTTCTACTACTCCAAC	21	35	24
bra-miR164b-3-p5	TGGAGAAGCAGGGCACGTGCG	21	8	60
bra-miR164b-3-p3	CACGTGTTCTACTACTCCAAC	21	35	24
bra-miR164c-p5	TGGAGAAGCAGGGCACGTGCAT	22	9	12
bra-miR164c-p3	CACGTGCTCCCCTCCTCCAAC	21	41	19
bra-miR164d-p5	TGGAGAAGCAGGGCACGTGCAC	22	7	2
bra-miR164d-p3	CACGTGCTCCCCTCCTCCAAC	21	41	19
bra-miR172c-p5	GCAGCATCATTAAGATTCACA	21	0	3
bra-miR172c-p3	GGAATCTTGATGATGCTGCAT	21	52	106
bra-miR172d-p5	GCATCATCATCAAGATTCAGA	21	8	47
bra-miR172d-p3	AGAATCTTGATGATGCTGCAG	21	135	633
bra-miR2111c-p5	TAATCTGCATCCTGGGGTTTA	21	0	1
bra-miR2111c-p3	GACCTCAGGATGCGGATTACC	21	3	4
