# Supplementary figures and images for: Identification of novel and conserved miRNAs involved in pollen development in Brassica campestris ssp. chinensis by high-throughput sequencing and degradome analysis
Source: BMC Genomics. 2014 Feb 21;15:146. doi: 10.1186/1471-2164-15-146 (PMC3936892; doi:10.1186/1471-2164-15-146)

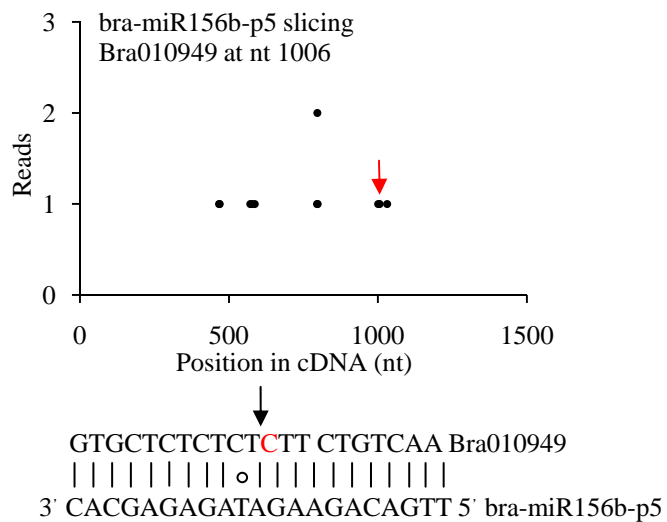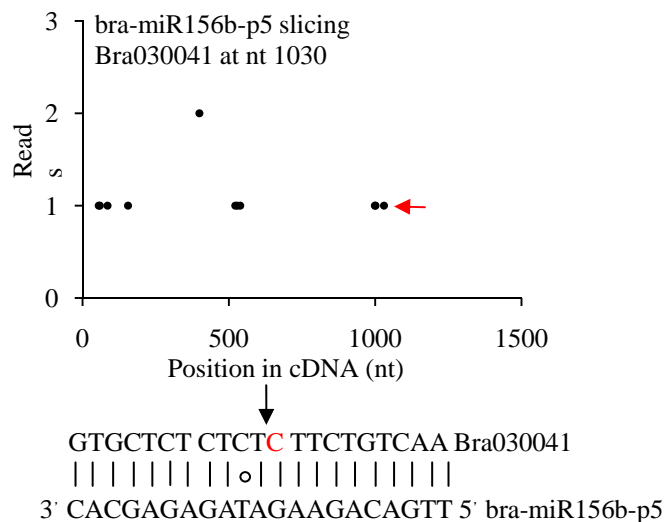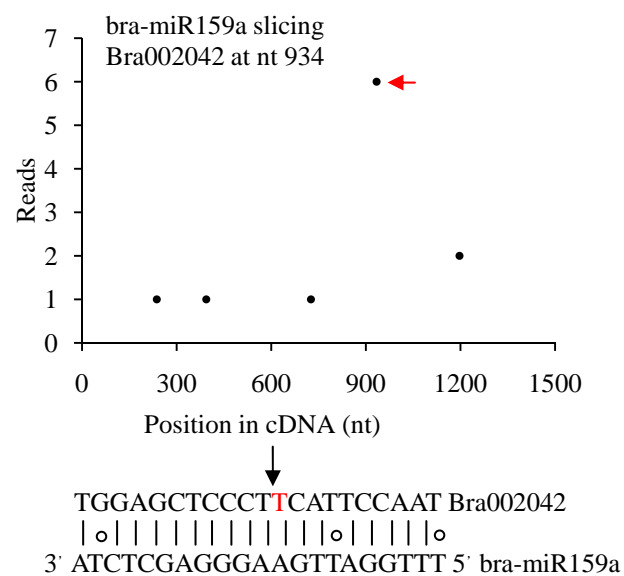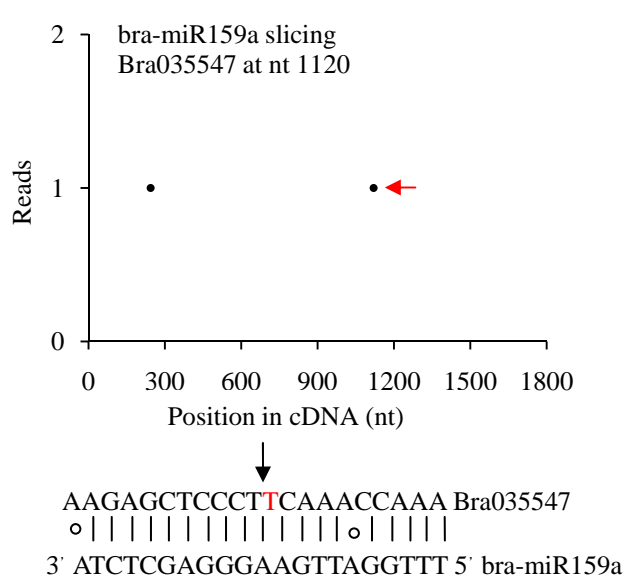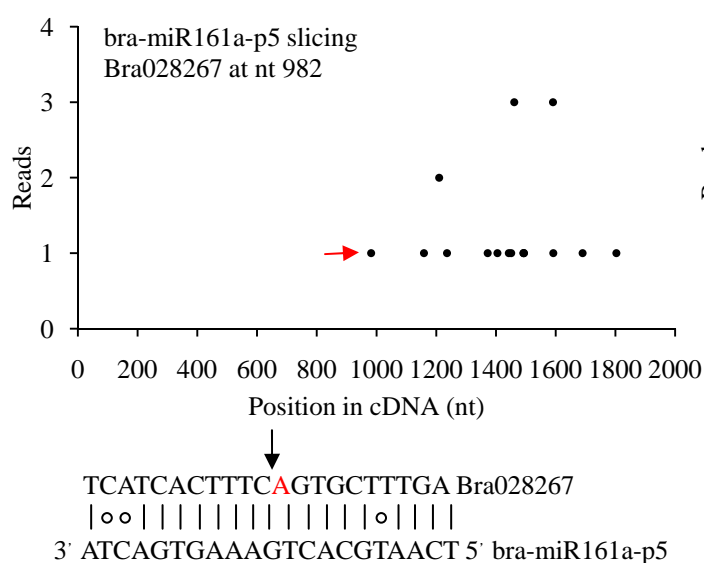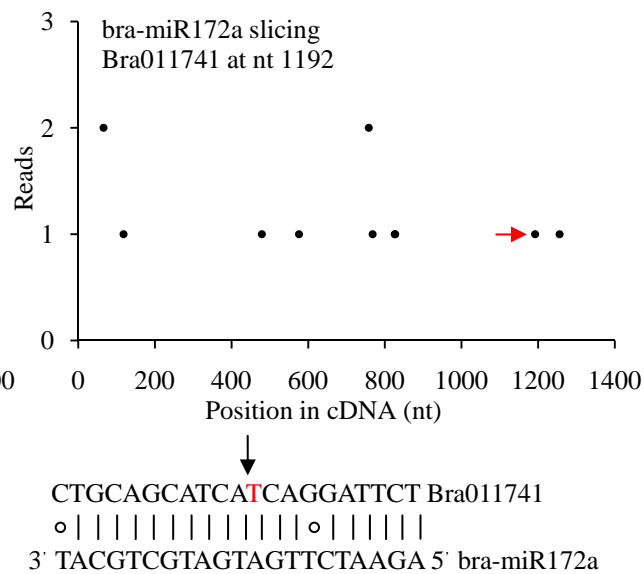

Supplement: Additional file 3: Figure S1 — Target plots (t-plots) of miRNAs targets confirmed by using degradome sequencing in Brassica campestris. [file 1471-2164-15-146-S3.pdf]
